# Synthesis of Alginate-Curcumin Nanocomposite and Its Protective Role in Transgenic *Drosophila* Model of Parkinson's Disease

**DOI:** 10.1155/2013/794582

**Published:** 2013-09-19

**Authors:** Yasir Hasan Siddique, Wasi Khan, Braj Raj Singh, Alim H. Naqvi

**Affiliations:** ^1^Drosophila Transgenic Laboratory, Section of Genetics, Department of Zoology, Faculty of Life Sciences, Aligarh Muslim University, Aligarh 202002, India; ^2^Centre of Excellence in Materials Science (Nanomaterials), Department of Applied Physics, Z.H. College of Engineering & Technology, Aligarh Muslim University, Aligarh 202002, India

## Abstract

The genetic models in *Drosophila* provide a platform to understand the mechanism associated with degenerative diseases. The model for Parkinson's disease (PD) based on normal human alpha-synuclein (**α**S) expression was used in the present study. The aggregation of **α**S in brain leads to the formation of Lewy bodies and selective loss of dopaminergic neurons due to oxidative stress. Polyphenols generally have the reduced oral bioavailability, increased metabolic turnover, and lower permeability through the blood brain barrier. In the present study, the effect of synthesized alginate-curcumin nanocomposite was studied on the climbing ability of the PD model flies, lipid peroxidation, and apoptosis in the brain of PD model flies. The alginate-curcumin nanocomposite at final doses of 10^−5^, 10^−3^, and 10^−1^ g/mL was supplemented with diet, and the flies were allowed to feed for 24 days. A significant dose-dependent delay in the loss of climbing ability and reduction in the oxidative stress and apoptosis in the brain of PD model flies were observed. The results suggest that alginate-curcumin nanocomposite is potent in delaying the climbing disability of PD model flies and also reduced the oxidative stress as well as apoptosis in the brain of PD model flies.

## 1. Introduction

Curcumin, a polyphenol extracted from the herb *curcuma longa* L., has been reported to possess antigenotoxic, anticancer, anti-inflammatory, antioxidant, antitumor, and several other biological as well as pharmacological activities [1, 2]. However, the retention time of the curcumin in body is limited due to its rapid systemic elimination and therefore restricts the therapeutic efficacy of curcumin [3]. In addition to the above properties, it has been reported to slow down the progress of Alzheimer's disease by reducing amyloid-*β* [4]. It delayed the onset of kainic acid-induced seizures [5]. Nanotechnology is the powerful tool for creating new objects in nanoscale dimensions having important applications in modern biomedical research [6]. Nowadays, ways to get therapeutic drugs to the central nervous system (CNS) effectively, safely, and conveniently are becoming more important than ever. The biomedical and pharmaceutical applications of nanotechnology have greatly facilitated the diagnosis and treatment of CNS diseases [7]. Nanoparticles can be utilized to maintain drug levels in a therapeutically desirable range with longer half-lives, solubility, stability and permeability [7]. In this context, the alginate-curcumin nanocomposite was synthesized, and its effect was studied on the climbing ability, lipid peroxidation, and apoptosis in the brain of PD model flies exhibiting human alpha-synuclein *α*S in the neurons.

## 2. Materials and Methods

### 2.1. Synthesis of Alginate-Curcumin Nanocomposite

The curcumin (100 mg, 0.27 mmol) was dissolved in the 20 mL dichloromethane, and about 2 mL of this solution was added dropwise into the warm 0.10% (100 mL) aqueous solution of sodium alginate. The mixture was ultrasonicated at 100 W with 30 kHz frequency for 10 min and heated at 50°C for 30 min. The obtained alginate-curcumin nanocomposite mixture was air-dried. The dried alginate-curcumin nanocomposite powder was stored in amber color vials at room temperature under dry and dark conditions until used for further characterization.

### 2.2. Characterization of Alginate-Curcumin Nanocomposite

#### 2.2.1. X-Ray Diffraction (XRD)

The X-ray diffraction (XRD) pattern of dried alginate-curcumin nanocomposite powder sample was recorded on MiniFlex II benchtop XRD system (Rigaku Corporation, Tokyo, Japan) operating at 30 kV and a current of 15 mA with Cu *K*_*α*_ radiation (*λ* = 1.5418 Å). The diffracted intensities were recorded in the 2*θ* angles from 20° to 80°. The crystallite size (*D*) of the curcumin in nanocomposite was calculated by using the Debye-Scherrer formula; that is, *D* = 0.9*λ*/*β* cos⁡*θ*, where *λ* is the wavelength of X-rays, *β* is the broadening of the diffraction line measured at half of its maximum intensity in radians, and *θ* is the Bragg's diffraction angle. The crystallite size of the curcumin nanoparticles embedded in the alginate-curcumin nanocomposite was determined by employing the full width at half maximum (FWHM) value of the most intense XRD peak. 

#### 2.2.2. Fourier Transform Infrared (FTIR) Spectroscopy

For the FTIR spectroscopic measurements alginate-curcumin nanocomposite powder was mixed with spectroscopic grade potassium bromide (KBr) in the ratio of 1 : 100, and spectra were recorded in the range of 400–4000 cm^−1^ wavenumber on Perkin Elmer FTIR Spectrum BX (Perkin Elmer Life and Analytical Sciences, CT, USA) in the diffuse reflectance mode at a resolution of 4 cm^−1^ in KBr pellets.

#### 2.2.3. AFM Analysis of Alginate-Curcumin Nanocomposite

The thin film of the alginate-curcumin nanocomposite was prepared on the borosilicate glass slide for the analysis of surface morphology. The prepared thin film was analyzed on the Atomic Force Microscope (AFM; Innova SPM, Veeco) in tapping mode. The commercial etched silicon tips as scanning probes with typical resonance frequency of 300 Hz (RTESP, Veeco) were used. The microscope was placed on a HOLMARC antivibration desk, under a damping cover. The processing was conducted using the SPM Lab software, and the scanning size was set at 0.1 *μ*m × 0.1 *μ*m. 

### 2.3. *Drosophila* Stocks

Transgenic fly lines that express wild-type h-*α*S under UAS control in neurons “(w[∗]; p{w[+mC] = UAS-Hsap/SNCA.F}” 5B and GAL4 “w[∗]; P{w[+mC] = GAL4-elavL}3)” were purchased from Bloomington *Drosophila* stock centre (Indiana University, Bloomington, IN). When the males of upstream activation sequence (UAS)-Hsap/SNCA.F strains are crossed with the females of GAL4-elav.L (vice versa), the progeny will express the human *α*S in the neurons [8]. The GAL4-UAS system is used to study gene expression and function in organism such as the fruit fly. The system has two parts: the GAL4 gene, encoding the yeast transcription activator protein GAL4, and the UAS, a short section of the promoter region, to which Gal4 specifically binds to activate gene transcription [9].

### 2.4. *Drosophila* Culture and Crosses

The flies were cultured on standard *Drosophila* food containing agar, corn meal, sugar, and yeast at 25°C (24 ± 1) [10]. Crosses were set up using six virgin females of UAS-Hsap/SNCA.F5B mated to three males of GAL4-elav. The progeny expresses the h-*α*S in the neurons, and the flies were referred as PD flies. All the assays were performed only on male flies. UAS-Hsap/SNCA.F flies were taken as a control in all the assays. The PD flies without any treatment were the positive control. The PD flies were also exposed separately to different doses of alginate-curcumin nanocomposite (ACNC) mixed in the culture medium. ACNC was added in the medium at final concentrations of 10^−5^, 10^−3^, and 10^-1 ^g/mL. As a negative control, the PD flies were allowed to feed on the diet supplemented with 10^-3 ^M of dopamine. The control flies (UAS-Hsap/SNCA.F) were also exposed to the selected doses of ACNC for the PD flies to see any negative effects.

### 2.5. *Drosophila* Climbing Assay

 The climbing assay was performed as described by Pendleton et al. [11]. Ten flies were placed in empty glass vials (10.5 cm × 2.5 cm). A horizontal line was drawn 8 cm above the bottom of the vial. After the flies had acclimated for 10 min at room temperature, both controls and treated groups were assayed at random, to a total of 10 trials for each. The procedure involved gently tapping the flies down to the bottom of the vials. The number of flies above the mark of the vial was counted after 10 s of climbing and repeated for 10 times to get the mean number above the mark of flies in this vial. These values were then averaged, and a group mean and standard error were obtained. All behavioral studies were performed at 25°C under standard lighting conditions.

### 2.6. Lipid Peroxidation Assay

 Lipid peroxidation assay in the brain homogenate was performed according to the procedure described by [12]. Reagent 1 (R1) was prepared by dissolving 0.064 g of 1-methyl-2-phenylindole into 30 mL of acetonitrile to which 10 mL of methanol was added to bring the volume to 40 mL. The preparation of 37% HCl served as the reagent R2. The brain of flies were isolated under stereozoom microscope in an ice cold Tris HCl (20 mM) (10 brain/group; five replicates/group). Homogenate was prepared in Tris HCl and centrifuged at 3000 g for 20 min, and subsequently the supernatant was collected. In a microcentrifuge tube 1300 *μ*L of R1 was taken. A volume of 1 *μ*L of supernatant was diluted 10 times with Tris HCl, and 200 *μ*L of this diluted supernatant from each group was added to 200 *μ*L of distilled water and vortex. Then 300 *μ*L of R2 was added to each tube, which was then vortexed and incubated at 45°C for 40 min. After incubation, the tubes were cooled in ice and centrifuged at 15000 g for 10 min at 4°C. All samples were read at 586 nm. 

### 2.7. Analysis of Cell Death in the *Drosophila* Brain

 The cell death in the *Drosophila* brain was analyzed as per the method described by [13]. Flies (5/treatments; 5 replicates) were placed in 70% ethanol in a 2 mL microcentrifuge tube for a minute. After 24 days of exposure to various doses of ACNC the brain was isolated in Ringer's solution under stereozoom microscope. After removing the Ringer's solution, about 100 *μ*L of freshly prepared acridine orange (5 *μ*g/mL) was added for 5 minutes. The brain was rinsed by Ringer's solution and immediately viewed and photographed through fluorescent microscope (OPTIKA, Italy). The image analysis program Image J (available online at http://rsb.info.nih.gov/ij/) was used to analyze the greyscale values for each brain.

### 2.8. Statistical Analysis

 The statistical analyses were done using Statistica Soft Inc. The student's *t*-test was applied to observe the significant difference between treatments and controls.

## 3. Results 

**Figure 1:**
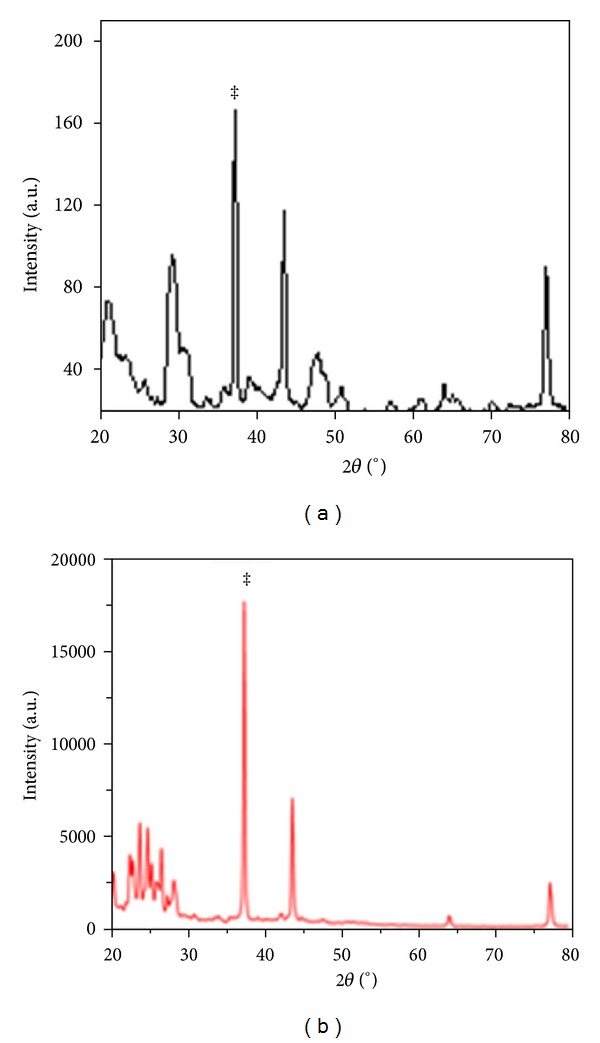
XRD pattern of alginate-curcumin nanocomposite shows the crystalline nature of curcumin nanoparticles (a) and curcumin (b). The Bragg reflection peak shown by the asterisk was used to calculate the crystalline size of the curcumin nanoparticles.

**Figure 2:**
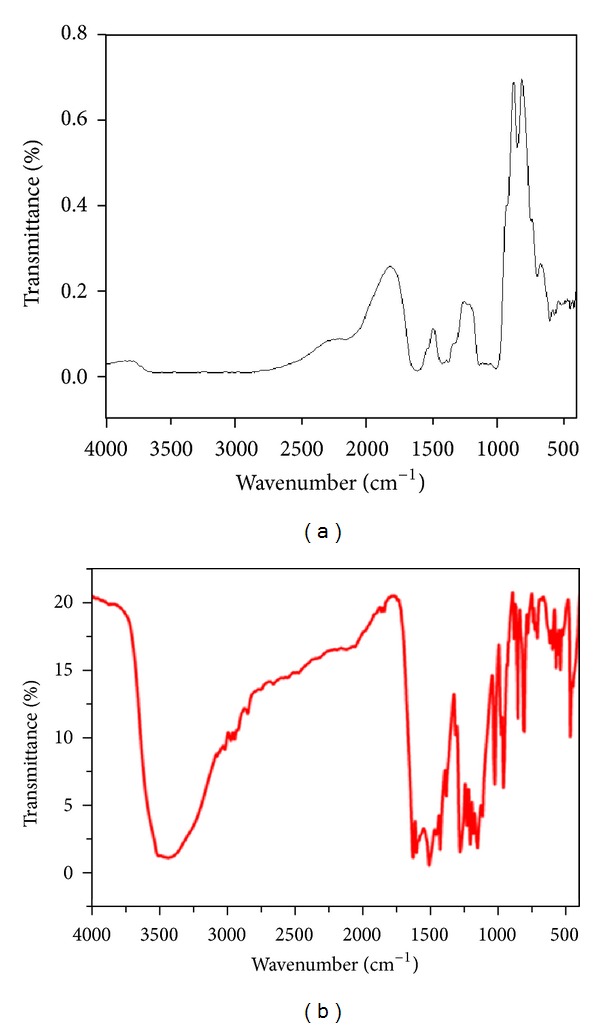
FTIR spectrum of alginate-curcumin nanocomposite shows the conjugation of the curcumin nanoparticles with the alginate (a) and curcumin (b).

**Figure 3:**
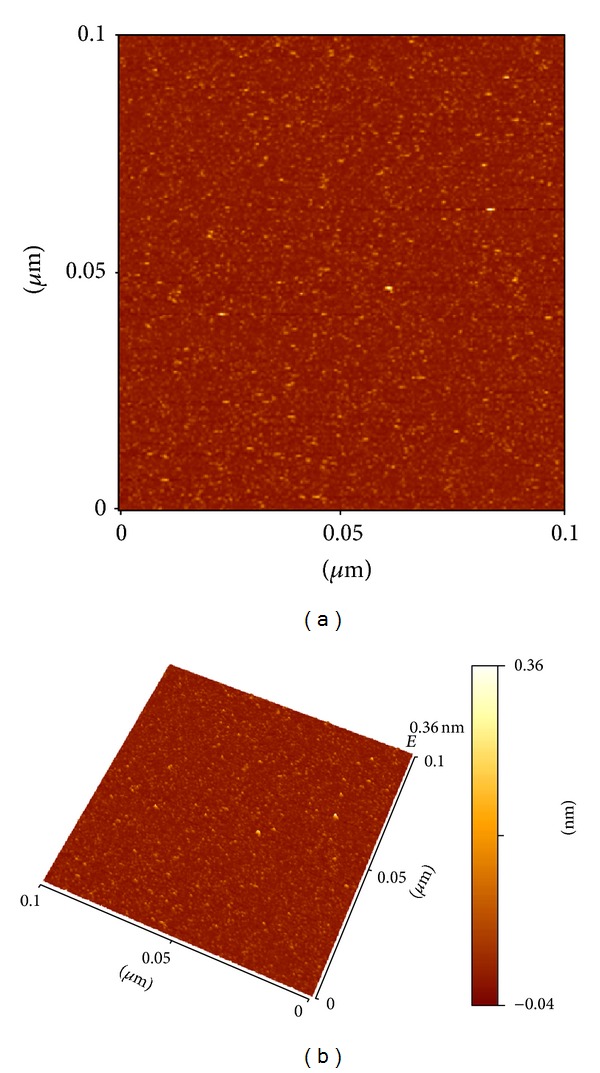
Atomic force micrographs in 2D (a) and 3D (b) illustrate the nanostructure of synthesized alginate-curcumin nanocomposite, respectively.

**Figure 4:**
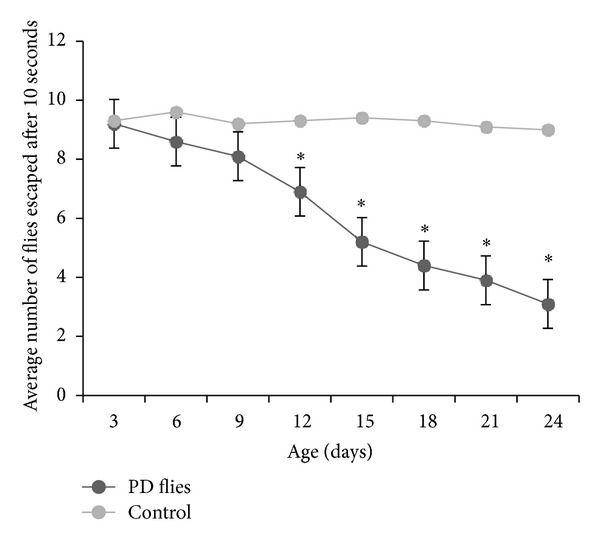
Climbing ability in Parkinson's disease (PD) flies and control for a period of 24 days. The values are mean of five assays (*significant with respect to control *P* < 0.01).

**Figure 5:**
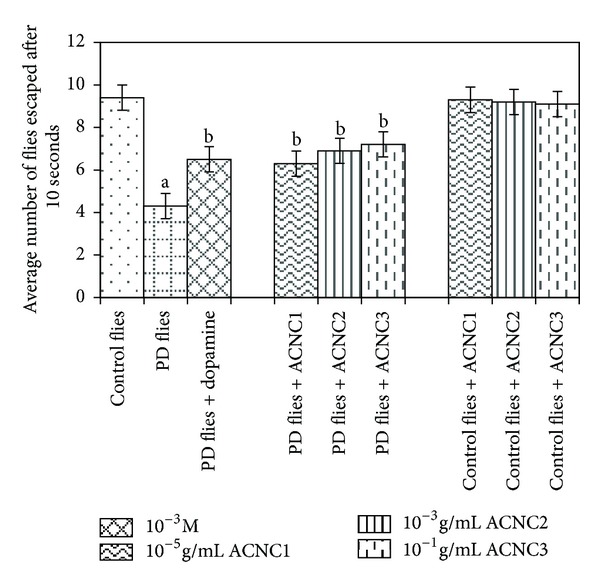
Effect of alginate-curcumin nanocomposite (ACNC) on climbing ability. The flies were allowed to feed on the diet supplemented with alginate-curcumin nanocomposite for 24 days and then assayed for climbing ability. The values are the mean of five assays (^a^significant with respect to control, *P* < 0.01; ^b^significant with respect to PD flies, *P* < 0.05).

**Figure 6:**
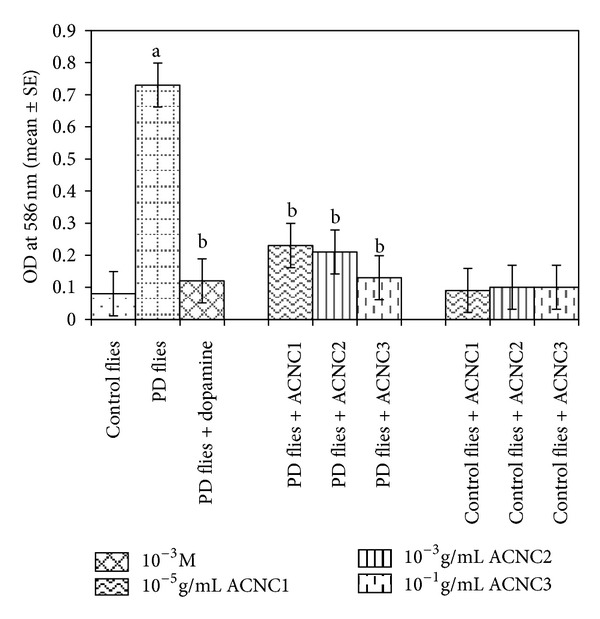
Effect of alginate-curcumin nanocomposite on the lipid peroxidation in the brain of PD flies. The flies were allowed to feed on the diet supplemented with alginate-curcumin nanocomposite for 24 days and then assayed for lipid peroxidation. The values are the mean of five assays (^a^significant with respect to control, *P* < 0.01; ^b^significant with respect to PD flies, *P* < 0.05).

**Figure 7:**
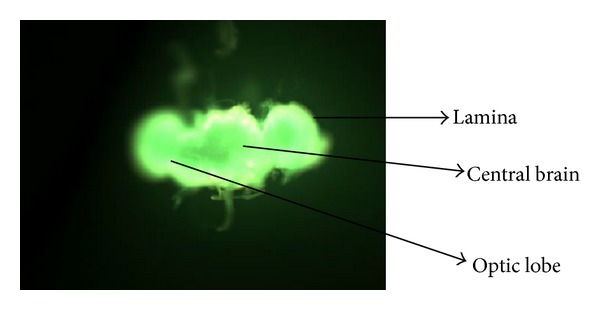
Transgenic* Drosophila melanogaster* brain stained by acridine orange.

**Figure 8:**
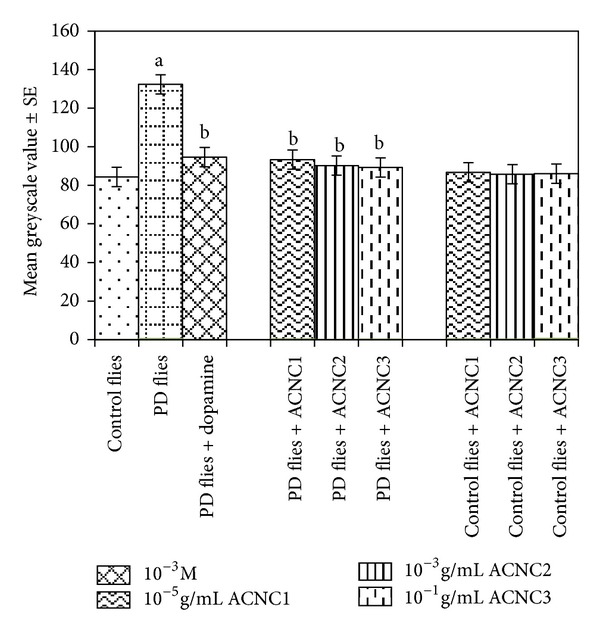
Effect of alginate-curcumin nanocomposite (ACNC) on the mean greyscale value. The flies were allowed to feed on the diet supplemented with alginate-curcumin nanocomposite for 24 days and then assayed for mean greyscale value. The values are the mean of five assays (^a^significant with respect to control, *P* < 0.01; ^b^significant with respect to PD flies, *P* < 0.05).

The XRD pattern revealed the crystalline nature of the curcumin embedded in the alginate-curcumin nanocomposite as shown in Figure 1. The full width at half maximum (FWHM) value for most intense peak was used to calculate the size of the curcumin nanoparticles. The average particle size of the curcumin in the alginate-curcumin nanocomposite was found to be ~11.3 nm (Figure 1). FTIR spectrum was analyzed to characterize the potential interactions alginate and curcumin nanoparticles embedded in the alginate-curcumin matrix. FTIR spectrum of alginate-curcumin nanocomposite is shown in Figure 2. The spectrum of alginate-curcumin nanocomposite analysis revealed that symmetric 1601 cm^−1^ to 1632 cm^−1^ and asymmetric 1407 cm^−1^ to 1421 cm^−1^ stretching vibrations of COO– group's peaks were shifted, respectively. The saccharide C-O-C stretching band of alginate was also shifted from the 1026 cm^−1^ to 1051 cm^−1^ in the spectrum of alginate-curcumin nanocomposite. Together the data demonstrated that the shifting of the alginate functional group peaks was attributed due to the conjugation of curcumin nanoparticles with the alginate matrix [14].  Figure 3 displays the AFM images of alginate-curcumin nanocomposite particles in 2D and 3D views. It is clear that particles have smooth and spherical morphology whose size lies in the range of ~10–19 nm. These results are consistent with XRD data. The results confirmed that the alginate-curcumin nanocomposite was successfully synthesized. The climbing response of control flies remained essentially unchanged over 24 days in a time course evaluation (Figure 4). From the day 12 on, however, the responses of the PD flies were significantly lower than those of the control. Based on these results, 24 days as standard duration of treatment was selected for the subsequent treatments with various doses of ACNC. The climbing assay was performed after 24 days of the exposure to various doses of ACNC. The exposure of PD flies to 10^-5 ^g/mL of ACNC showed a significant delay in the loss of climbing ability (Figure 5). Similarly, the exposure of PD model flies to 10^−3^ and 10^-1 ^g/mL of ACNC also showed a significant delay in the loss of climbing ability (Figure 5). A significant increase in the lipid peroxidation was observed in the brain of PD model flies (0.73 ± 0.008) as compared to control flies (0.08 ± 0.001). A significant dose-dependent decrease in the lipid peroxidation was observed when the PD model flies were exposed to 10^-5 ^g/mL (0.23 ± 0.002), 10^-3 ^g/mL (0.21 ± 0.001), and 10^-1 ^g/mL (0.13 ± 0.001) doses of ACNC added in diet (Figure 6). For the apoptotic analysis the mean greyscale values were calculated for the brains of flies for each treatment.  Figure 7 shows the isolated brain for *Drosophila *stained with acridine orange. The brain of control flies was associated with the mean greyscale value of 84.3653 ± 0.3463. For the PD model flies the mean greyscale value was 132.3857 ± 0.7213. The exposure of PD model flies to 10^−5^, 10^−3^, and 10^-1 ^g/mL of ACNC was associated with mean greyscale value of 93.3480 ± 0.6134, 90.2163 ± 0.3311, and 89.3216 ± 0.5213, respectively (Figure 8).

## 4. Discussion

Alpha-synuclein is a presynaptic protein that is expressed at synaptic terminals in the CNS [15, 16]. In its native form it is unfolded protein, but the monomeric (native) forms can misfold and aggregate into a larger oligomeric-fibrillar forms and result in the formation of most neurotoxic species [17, 18]. The generation of reactive oxygen species (ROS) has been correlated with onset of PD; the polyphenolic compounds having antioxidant potential may have therapeutic value. Curcumin has been reported to reduce the inflammation and oxidative damage in Alzheimer's disease (AD) [19, 20]. It showed protective effect against *α*S-induced cytotoxicity in SH-SY5Y neuroblastoma cells by decreasing cytotoxicity of aggregated *α*S, reducing intracellular ROS, inhibiting caspase-3 activation and ameliorating the signs of apoptosis [21]. Intracellular as well as extracellular addition of oligomeric *α*S has been reported to increase the generation of intracellular ROS in SH-SY5Y cells [21]. The exposure of PD model flies to different doses of alginate-curcumin showed reduction in the lipid peroxidation in the brain of PD model flies, which shows the antioxidant potential of alginate-curcumin nanocomposite. Lipid or polymer based nanoparticles can be designed to improve the pharmacological and therapeutic properties of the drugs [22]. The blood brain barrier (BBB) is a separation of circulating blood from the brain extracellular fluid (BECF) in the CNS. Because of the tight junctions, the BBB allows only highly lipid soluble molecules under a threshold of 400–600 Daltons to penetrate [23, 24]. A wide range of CNS drugs including large molecular weight biological therapeutic peptides, proteins, and genes may gain entry into the brain with nanoparticles as carriers [7]. The fly has a blood brain barrier (BBB) and an immune system; however, these are simple in comparison with their mammalian counterparts, suggesting that diseases associated with neuroinflammation will be difficult to study in model flies and that neuroprotective compounds studied in *Drosophila* may need to be altered to pass a mammalian BBB [25]. The alginate-curcumin nanocomposite used in the present study shows the protective effect against the progression of PD symptoms in model flies. Ligands with multiple receptor binding sites (multivalent) can crosslink the membrane receptors more efficiently to regulate signalling process. Hence by changing the size, shape, and material properties of engineered nanoparticles, the degree of receptor crosslinking and subsequently cell responses can be precisely controlled [26]. The results of the present study reveal that the alginate-curcumin nanocomposite (ACNC) significantly delayed the loss of climbing ability of the PD model flies and reduced oxidative damage as well as apoptosis in the brain of PD model flies. The synthesis of ACNC reported in this study is based on a wet-milling technique [27] which involved the addition of the desired concentration of curcumin solution in a volatile organic solvent into hot water under specific ultrasonic conditions. The superior aqueous solubility of alginate-curcumin nanocomposite could be attributed to the larger surface area of the curcumin nanoparticles as compared to the bulk curcumin [28, 29]. The selective loss of dopaminergic neurons in the substantia nigra pars compacta leads to the reduction of dopamine content. The formation of Lewy bodies in surviving dopaminergic neurons is a pathological hallmark of this disorder [30]. The progression of PD is attributed to abnormal protein aggregation, oxidative damage, and mitochondrial dysfunction [31]. It thus appears that the conditions for *α*S to aggregate and form fibrils, such as, increase in gene copy number [32] missense mutation [33] oxidative modification [34] phosphorylation [35] the presence of metal ions [36] and interaction with phospholipid membranes [37] leads to the pathogenesis of the PD [38]. Besides having the therapeutic approaches of increasing dopaminergic neurons activity or inhibiting the cholinergic effects to the striatum, nowadays the attention has also been given to the use of flavonoids/natural antioxidants to reduce the oxidative stress [39–42]. Various flavonoids have been reported to act as molecular inhibitors of *α*S aggregation and therefore could act as possible protective agents against the progression of the PD. Recent findings suggest that flavonoids have a remodeling effect on the nature of *α*-synuclein fibrils, converting them into nontoxic, smaller amorphous aggregates [43]. Polyphenols generally show the reduced oral bioavailability, increased metabolic turnover, and lower permeability through the blood brain barrier [44]. As a result for the maintenance of high concentration in plasma, the repeated ingestion of polyphenols has been suggested [38]. Oxidative stress is one of the important factors in PD as a result of the destructive effect of free radicals [45–47]. Free radical scavengers have been reported to prevent or reduce the rate of progression of the PD [48, 49]. Current therapeutic strategies rely on providing protection against the massive degenerative loss of dopamine neurons, particularly in the substantia nigra. The efficacy of levodopa declines as PD progresses, but the oxidative stress can be reduced by agents having the free radical scavenging potential [50]. Our earlier studies with nordihydroguaiaretic acid [51], curcumin [52], capsaicin [53], and ascorbic acid [54] also showed a dose-dependent delay in the loss of climbing ability in the same PD model flies. The study with ascorbic acid, a well known antioxidant that showed no significant difference in the protein levels in the brain of PD model fly, suggests that only free radical scavenging activity is involved in the protection against the PD symptoms [54]. The decrease in lipid peroxidation and apoptosis in the brain of PD model flies may be due to the inhibitory effect of the ACNC on *α*S aggregation or due to the scavenging of free radicals resulting from the oxidative stress. 

Acridine orange is a vital dye that specifically stains cells undergoing apoptosis in *Drosophila melanogaster*. It has been shown to be specific for apoptotic cells and does not stain the chromatin of cells dying by oxygen starvation or necrosis [55]. The increased levels of apoptosis are reflected by increasing fluorescence [13]. When stained with acridine orange, cell undergoing apoptosis will show bright fluorescence under fluorescent microscopy. The image analysis program Image J can be used for calculating the greyscale values for each brain. A more intensity fluorescent brain would possess a higher average greyscale value as compared to a less intensity fluorescing brain. The results obtained in our present study by using Image J area calculator showed that the average greyscale value was highest for the PD model flies and the exposure of PD model flies to values doses, that is, 10^−5^, 10^−3^, and 10^-1 ^g/mL of ACNC showed a dose-dependent decrease in the average greyscale values, suggesting that the exposure of ACNC prevents the brain cells, possibly by reducing the oxidative stress as is evident by the reduction in the lipid peroxidation in the brain of PD model flies. 

## 5. Conclusion

Due to ethical issues, most of the studies have been carried out on model organisms, including mice, fruit flies, worms, and cell lines. The European Centre for the Validation of Alternative Methods (EVCAM) has recommended the use of *Drosophila* as an alternative model for scientific studies [56, 57]. The results in the present study suggest that the transgenic fly model mimics the motor impairments associated with PD, and a climbing assay can be performed to determine whether or not a variety of compounds or drugs mixed in the fly culture medium prevent the progressive loss of climbing ability [11]. Although there are reports that curcumin crosses the blood brain barrier and is neuroprotective in neurological disorders [58], the nanoparticles have increased half-lives, solubility, and stability [7]. Presently nanotechnology is revolutionizing development of drug delivery, imaging, and diagnosis. Due to the inherent complexity of the CNS, the nanoparticles showing promising results in the treatment of PD should be further scrutinized prior to their clinical applications.
